# Dithiol Based on l-Cysteine and Cysteamine as a Disulfide-Reducing Agent

**DOI:** 10.1021/acs.joc.2c01050

**Published:** 2022-07-21

**Authors:** Francesca Bartoccini, Michele Retini, Rita Crinelli, Michele Menotta, Alessandra Fraternale, Giovanni Piersanti

**Affiliations:** Department of Biomolecular Sciences, University of Urbino Carlo Bo, Piazza Rinascimento 6, 61029 Urbino, PU, Italy

## Abstract

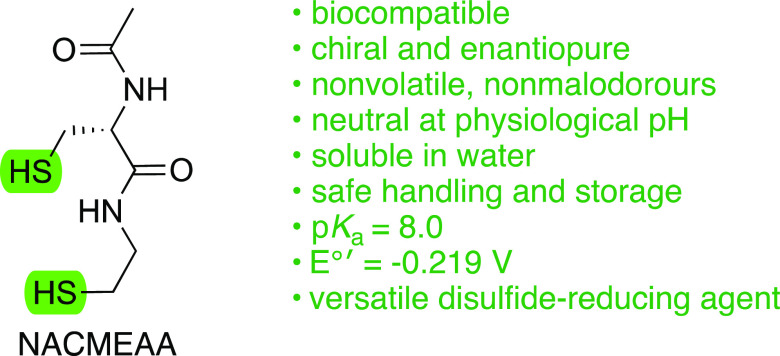

We report the synthesis, chemical properties, and disulfide bond-reducing performance of a dithiol called NACMEAA, conceived as a hybrid of two biologically relevant thiols: cysteine and cysteamine. NACMEAA is conveniently prepared from inexpensive l-cystine in an efficient manner. As a nonvolatile, highly soluble, and neutral compound at physiological pH with the first thiol p*K*_a_ value of 8.0, NACMEAA is reactive and user-friendly. We also demonstrate that NACMEAA reduces disulfide bonds in GSSG and lysozyme.

## Introduction

Since the first report on the preparation and use of a seminal synthetic reductive dithiol, racemic (2*S*,3*S*)-1,4-bis(sulfanyl)butane-2,3-diol (dithiothreitol or DTT, Table 1), by Cleland in 1964,^1^ the development of dithiols has become a field of great interest for a broad number of applications, with many groups involved worldwide.^2^ The continuing interest in the development of superior and practical dithiol systems stems from their ability to maintain thiols completely in the reduced form and to reduce disulfide bonds that often confer their biomolecular function as well as their reactivity in cysteine-based bioconjugation chemistry.^3^ Dithiols have an advantage over monothiols (such as glutathione, cysteine, or cysteamine) in that the native trans-thiolation products can be rapidly cleaved by the formation of intramolecular disulfide bonds. Cleland designed a water-soluble (two hydroxyl and two thiol functional groups on only four carbon atoms) solid (mp: 41–44 °C) compound that adopts a stable six-membered cyclic structure in its oxidized form; it has become the gold standard disulfide-reducing agent for use in all fields of biomolecular science. However, DTT suffers from several challenges: (1) at neutral pH, its thiol groups are protonated and, thus, it has a low reactivity as a reducing agent;^4^ (2) it is unstable in a slightly basic solution and has a very short half-life of 1.4 h at pH 8.5;^5^ (3) it has the ability to chelate metals and generate H_2_O_2_ on exposure to air;^6^ and most importantly, (4) it is toxic.^7^ To overcome these limitations, in 1991, Whitesides *et al*. developed two bias/constrained achiral α,ϖ dithiols, *N*,*N*′-dimethyl-2-sulfanyl-*N*′-(2-sulfanylacetyl)acetohydrazide (*N*,*N*′-dimethyl-*N*,*N*′-bis(mercapto-acetyl)hydrazine or DMH, Table 1) and (2*S*,5*R*)-*N*,*N*,*N*′,*N*′-tetramethyl-2,5-bis(sulfanyl)hexanediamide (2,5-dimercapto-*N*′,*N*′,*N*′,*N*′-tetramethyladipamide or DTA, Table 1), which are prone to form cyclic disulfides when oxidized to produce six- to eight-membered rings, with the presence of electron-withdrawing groups to lower the thiol p*K*_a_.^8^ Based on a similar logic and characterized by the ability to effectively reduce disulfides efficiently at neutral pH with a reducing potential in the range of that of DTT, Raines *et al*. reported the most significant enhancement with the preparation of two nitrogen-containing water-soluble dithiols called (2*S*)-2-aminobutane-1,4-dithiol (dithiobutylamine or DTBA, Table 1), from aspartic acid, and [3-(sulfanylmethyl)pyrazin-2-yl]methanethiol (2,3-bis(mercaptomethyl)pyrazine or BMMP, Table 1), from 2,3-dimethylpyrazine.^9^ The first p*K*_a_ value of the sulfhydryl groups in all of these dithiols is between 8.2 and 7.6 (more than one unit lower than that of DTT), thereby making them better reductants at a lower pH while maintaining similar thermodynamic reduction potentials but with different kinetic properties and (unfavorable) Coulombic interactions as DTT. In addition, a dibenzyl derivative of DTBA that is soluble in organic solvents and more compatible with solid-phase synthesis was also reported recently by de la Torre *et al*.^10^ Notably, in all cases, to install the requisite sulfur functionalities with a double Mitsunobu reaction,^11^ the malodorous compound thioacetic acid was employed, i.e., the source of sulfur of the final thiols was external and non-naturally occurring.

**Table 1:**
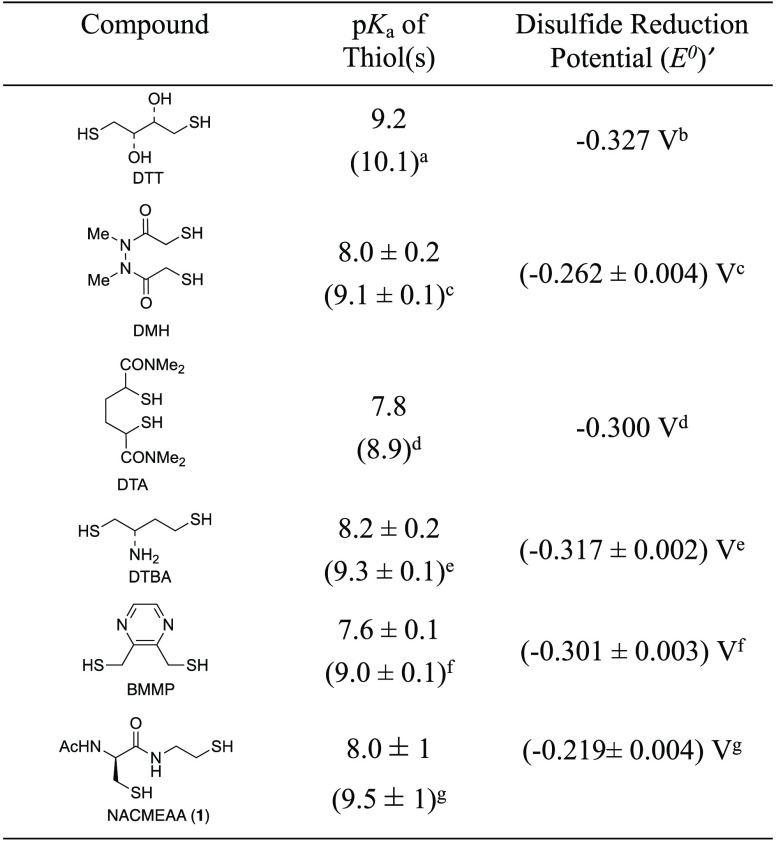
Physicochemical Properties of Dithiol-Reducing Agents

a Value is from ref (2a).

b Value is from ref (2b).

c Values are from ref (8a).

d Values are from ref (8b).

e Values are from ref (9a).

f Values are from ref (9b).

g Values are from this work.

Our goal was to identify an operationally convenient (nonmalodorous, nontoxic, neutral, and thermally stable), organic solvent- and water-miscible dithiol with a low p*K*_a_ and suitable disulfide redox potential, where the two sulfhydryl groups are derived from two of the most biologically relevant sulfur-containing compounds: l-cysteine and cysteamine. In addition, we reasoned that the presence of the central secondary amide bonded to the thiol-containing precursors could provide (a) an inductive effect for thiol acidity, (b) a hydrogen bond network for solubility and stability in water, (c) improvement in the biocompatibility, and (d) fluxionality to the switch between *trans*- and *cis*-amide conformers, which brings the sulfur atoms closer in space and promotes the formation of intramolecular disulfide bonds.^12^ We envisioned that (2*R*)-2-(acetylamino)-3-mercapto-*N*-(2-mercaptoethyl) propanamide (*N*-acetylcysteine mercaptoethylamine amide or NACMEAA (**1**)) could satisfy these criteria and be synthesized from abundant and largely accessible l-cystine, which indeed is produced via fermentation.^13^ Thus, as part of our ongoing interest in the modulation of redox signaling in cells,^14^ in this paper, we report the synthesis, physicochemical properties, and disulfide bond-reducing performance on both small molecules and biomolecules of this novel biocompatible reagent.

## Results and Discussion

**Scheme 1:**
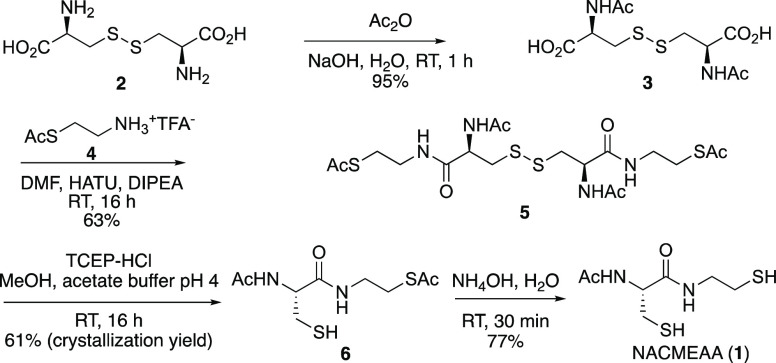
Four-Step Synthesis of NACMEAA

We accessed NACMEAA (**1**) via a new four-step route, depicted in Scheme 1, which avoids the use of flash chromatography purifications throughout the whole sequence and satisfies our aim of developing clean transformations and obtaining pure intermediates from simple recrystallizations and/or liquid separations.^15^ The route commenced with the double N-acetylation of l-cystine (**2**), an abundant and biobased raw material. Due to the low solubility of l-cystine (**2**) in common organic solvents and its high solubility in water at basic pH, we decided to treat l-cystine (**2**) with various acylating agents in basic aqueous solution at room temperature. Acetic anhydride, which is available in large quantities at reasonable prices, has proven to be an appropriate acetyl donor substrate, leading to product **3** in high yield after acidification with Dowex 50W-X8(H) and removal of water.^16^ The chemoselective formation of double amide bonds with *S*-acetyl cysteamine trifluoroacetate salt (**4**) turned out to be difficult, probably due to the thermodynamically favored S-to-N acyl transfer processes of the latter under basic conditions (see Table S1). Among the most reliable and widely used coupling reagent-mediated condensation methods to synthesize proteinogenic α-amino acid peptides, only the combination of HATU and a proper order of addition of the reagents (see below) efficiently provided a good yield and easy purification of **5**.^17^ When the same reaction conditions were applied to *N*-acetyl-cysteine or unprotected free cysteamine, the reaction was unsuccessful, highlighting the importance of having the thiol group protected/masked.^18^ The reductive cleavage of the disulfide bond with the commercially available and crystalline solid tris(2-carboxyethyl)phosphine hydrochloride (TCEP-HCl)^19^ in aqueous media yielded 2 equiv of thiol **6** in 61% yield after crystallization, which was subjected to mild thioester ammonolysis to give the desired NACMEAA (**1**) in 99% purity and an overall yield of 28%. The optical purity of these compounds was established by comparing the specific rotation values reported in the literature. (For example, NACMEAA showed [*α*]_D_^20^ = −55 (*c* = 0.24, CHCl_3_); lit.^15^ [*α*]_D_^20^ = −50 (*c* = 1.2, CHCl_3_)].) Reactions were typically carried out using 10 g of l-cystine but could be readily scaled up (see the Supporting Information). Unfortunately, performing the disulfide reduction with cheaper reducing agents such as zinc in acetic acid and magnesium in methanol was unsuccessful. Using sodium borohydride in THF or MeOH, very low conversion was observed after 48 h. When we treated **5** with 1.3 equiv of tributylphosphine, cheaper than TCEP-HCl albeit highly toxic with unpleasant smell, in MeOH/H_2_O (5:1), the formation of the product was detected together with the transposition products (see ref (15)). In addition, this reagent is more air-sensitive than TCEP, and the tributylphosphine oxide byproduct is hard to remove. TCEP has proven to be the most effective and practical reducing agent in this case, although it is quite expensive.^20^ Conversely, all the reactions were accomplished at room temperature, and three out of four steps were conducted in aqueous media. Aqueous systems for the amide coupling of **3** with **4** have also been briefly investigated using water, water–surfactant mixtures, and two-phase systems; however, none seemed to give acceptable results. Moreover, this synthetic strategy shows an increased efficiency compared to previous methods,^15^ which opens the door to further exploration of this scaffold. It is important to note that this approach may be more suitable for analog generation compared to previous methods.

NACMEAA (**1**) has desirable chemical properties. It is a practically odorless solid with a high solubility in water as well as in a broad range of organic solvents. It also has a low p*K*_a_. Using pH-titration experiments that were broad enough to cover the transition from protonated to unprotonated forms and were monitored by ultraviolet spectroscopy (absorbance at 238 nm),^21^ we determined the thiol p*K*_a_ values of NACMEAA to be 8.0 ± 0.1 and 9.5 ± 0.1 (Figure S1). Even though the p*K*_a_ value is not the main determinant for enhancing the reactivity of a thiol group and/or influencing the mechanisms of thiol–disulfide substitution, these values are lower than those of DTT and other similar dithiols reported previously (see Table 1).^22^

According to the ^1^H,^1^H-COSY spectrum, the most downfield-shifted resonance of NACMEAA belongs to the thiol proton of the cysteine part, suggesting that it is the most acidic proton (see Figure S5). Also, it is more acidic than cysteine itself (p*K*_a_ = 10.78), likely resulting from the inductive effects of the amido group and/or related to the engagement of the cysteinyl SH in an intramolecular H-bonding with the amide carbonyl; thus, NACMEAA has a more reactive thiolate population at physiological pH.^13^

**Figure 1:**
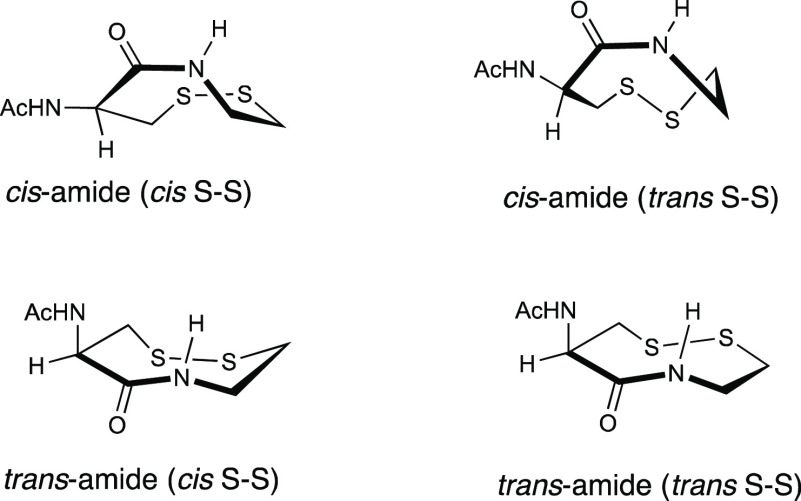
Conformations of **1**^ox^.

By equilibrating reduced NACMEAA with oxidized 2-mercaptoethanol (βME^ox^)^23^ and using HPLC to quantify reduced and oxidized species at pH 7, we found the reduction potential of oxidized NACMEAA (**1**^ox^) to be *E*°′ = (−0.219 ± 0.004) V (Figure S2). This value is within the range of disulfide redox potentials found in proteins but is close to the oxidizing end of the scale. We speculate that the relative instability of this disulfide bond is innate and is due to the strained nature of the eight-membered ring resulting from disulfide-bond formation. The ring strain is probably due to the distorted trans conformation that the ring adopts, as widely reported for similar vicinal disulfide ring-containing molecules (Figure 1).^24^ Moreover, considering that protein disulfide isomerase (PDI), the essential cellular enzyme that catalyzes the unscrambling of non-native disulfide bonds in other proteins, has an *E*°′ of −0.18 V, it could be an advantage in future PDI mimetic research.^25^ However, the value of *E*°′ for NACMEAA indicates that its ring closure is less favorable overall than that of DTT, so it is more resistant to oxidation on exposure to air.

**Figure 2:**
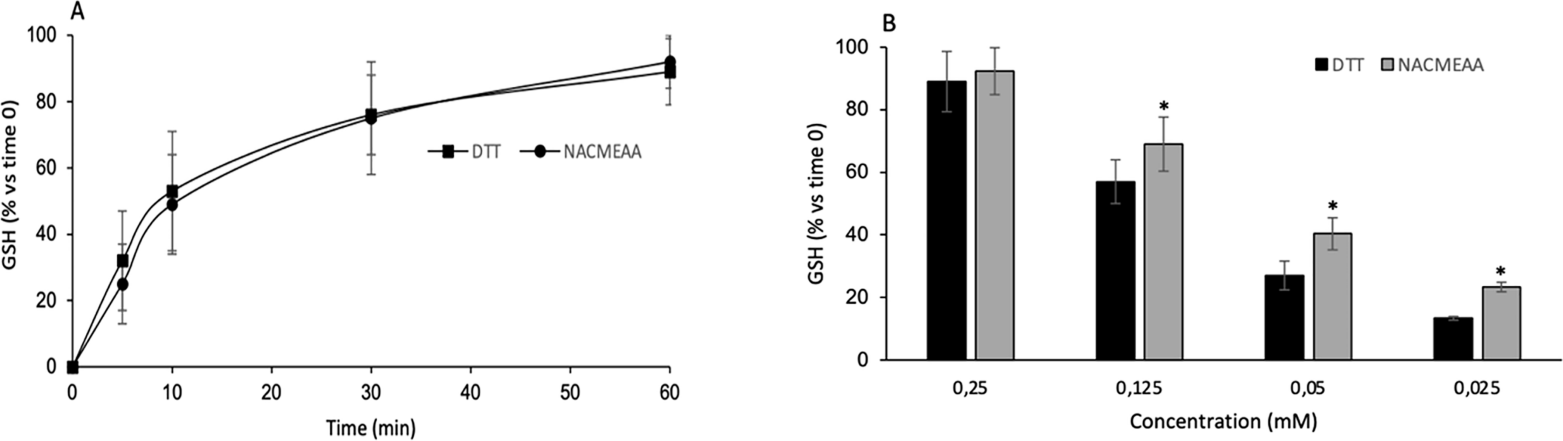
Oxidized l-glutathione (GSSG) reduction by DTT and NACMEAA. (A) Time course (5, 10, 30, and 60 min) of reduced glutathione (GSH) formation by incubation of 25 μM GSSG at pH 7.4 and 37 °C with 0.25 mM DTT or NACMEAA. (B) GSH formation after incubation of 25 μM GSSG in PBS at pH 7.4 and 37 °C for 1 h with different concentrations of DTT or NACMEAA. The results represent the mean ± SD of at least two independent experiments.

NACMEAA is an efficacious reducing agent for disulfide bonds. Time-course monitoring of oxidized l-glutathione reduction by 0.25 mM DTT vs NACMEAA showed comparable efficiencies of the two compounds (Figure 2), while higher GSH amounts were measured after 1 h of incubation with 0.025–0.125 mM NACMEAA (**1**), with respect to the same doses of DTT (Figure 2). The product of GSSG reduction, i.e., GSH, was determined by an HPLC method based on separation coupled with ultraviolet detection and precolumn derivatization with 5,5′-dithiobis-(2-nitrobenzoic acid).

**Figure 3:**
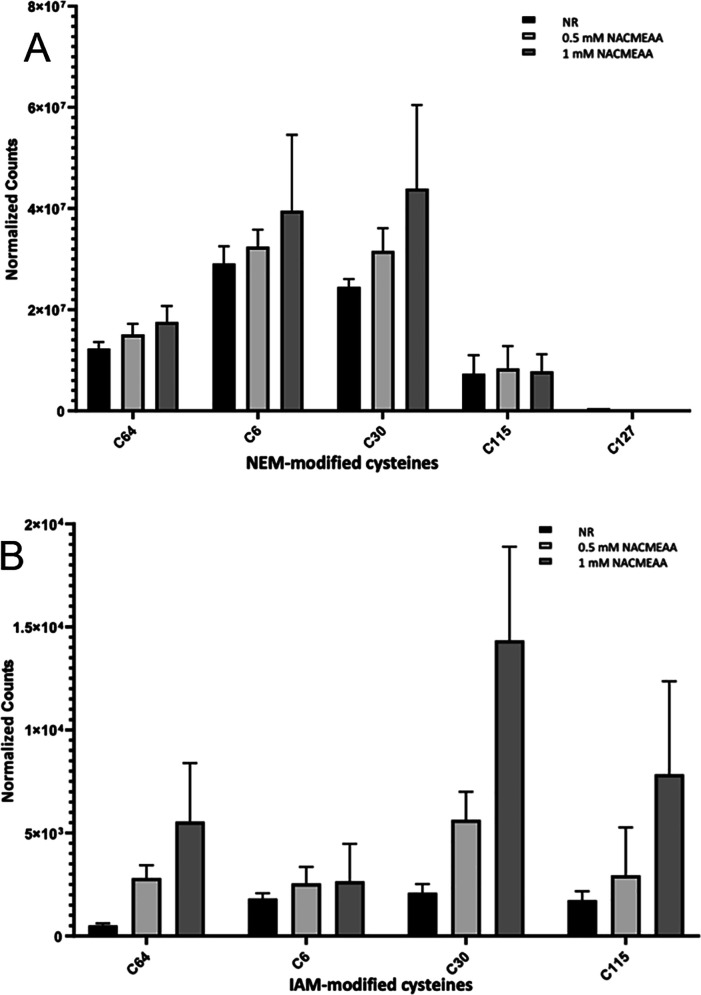
MS-identified peptides with cysteine modifications from lysozyme. Semiquantitative analysis of cysteine-containing peptides obtained in nontreated control lysozyme (NR) samples or samples of lysozyme treated with 0.5 or 1 mM NACMEAA and alkylated by (A) NEM or (B) IAM.

Based on the promising studies above, we then attempted to qualitatively identify protein disulfides that exhibit sensitivity to our dithiol-based reducing agents. Lysozyme, a mucolytic enzyme with antibiotic properties, is one of the most widely studied proteins and is often used as a model system to study the effect of additives on protein folding and aggregation.^26^ It is a typical globular protein that is comprised of an assortment of large and small α-helices and a few short β-sheets containing four disulfide bonds, two in the α-domain (Cys6–Cys127 and Cys30–Cys115), one in the β-domain (Cys64–Cys80), and another connecting the two domains (Cys76–Cys94).^27^ This protein was chosen to demonstrate the usefulness of NACMEAA as a valuable reducing agent for disulfide bonds in proteins. Furthermore, the microenvironment of each disulfide is very different in terms of its amino acid sequence and physicochemical properties. Lysozyme was subjected to a reduction–alkylation redox protocol (Figure 3).

Cysteines that undergo alkylation by iodoacetamide (IAM) or *N*-ethylmaleimide (NEM) after reduction with NACMEAA represent targets of these dithiol-based reducing agents (“redox-sensitive Cys”). Gratifyingly, the amount of NEM- or IAM-modified cysteines (Cys6, Cys30, Cys64, and Cys115) was increased in a concentration-dependent manner in all reduced lysozyme samples compared to the nonreduced (NR) samples, suggesting the ability of NACMEAA to reduce the disulfide bridge formed by these residues. The peptides containing alkylated cysteines at C76, C80, C94, and C127 were slightly or not detected, indicating that Cys76–Cys94, the interdomain disulfide, forms a stable bond or is buried in the protein core (Figure S3). Similar and rewarding results were also obtained with a larger globular plasma protein, bovine serum albumin,^28^ which contains eight disulfide bonds (and one free thiol group), during the evaluation and identification of redox-sensitive disulfides using 0.5 or 1 mM NACMEAA and IAM as the chemical probe for alkylation (Figure S4).

## Conclusions

In conclusion, we have developed a disulfide-reducing agent that exhibits favorable chemical and physical features. The two thiols from cysteine and cysteamine residues as well as the convoluted hydrogen-bonding networks provided by the two secondary amides allow NACMEAA to have good (bio)chemical attributes with a similar performance as DTT but with an extended pH range at which disulfide bonds can be efficiently reduced. These properties make NACMEAA an interesting reagent for the reduction of disulfide bonds, and we expect that it will complement existing methods in the field of chemical biology.

## Experimental Section

### General Methods

All reactions were run in air. Analytical thin-layer chromatography (TLC) was carried out on silica gel plates (silica gel 60 F_254_) that were visualized by exposure to ultraviolet light. The ^1^H NMR and ^13^C NMR spectra were recorded on a 400 spectrometer using CDCl_3_, CD_3_OD, and D_2_O as solvents. Chemical shifts (δ scale) are reported in parts per million (ppm) relative to the central peak of the solvent. Coupling constants (*J* values) are given in hertz (Hz). Structural assignments were made with additional information from the gCOSY experiment. Melting points were determined on a capillary melting point apparatus and were uncorrected. Optical rotation analysis was performed with a polarimeter using a sodium lamp (λ = 589 nm, D-line); [*α*]_D_^20^ values are reported in 10^–1^ deg cm^2^ g^–1^; concentration (*c*) is in g for 100 mL. HRMS analysis was performed using Orbitrap Exploris mass spectrometers.

### Starting Materials

l-Cystine (**2**), β-mercaptoethanol (βME), and 2-hydroxyethyldisulfide (βME_ox_) are commercially available. *S*-Acetyl cysteamine trifluoroacetate salt (**4**) was synthetized as reported in the literature.^29^

#### *N*,*N*′-Diacetyl-l-cystine (**3**)

To a suspension of l-cystine (**2**) (10 g, 41.7 mmol) in H_2_O (42 mL) were added 5 M NaOH (30 mL, 150 mmol) and acetic anhydride (12 mL, 127.2 mmol). The reaction mixture was stirred at room temperature for 90 min. The resulting crude was poured into a beaker containing Dowex 50W-X8(H) (100 mL of water-wet resin) and H_2_O (30 mL). The suspension was filtered, and the resin was washed with H_2_O (3 × 200 mL). The filtrate was concentrated at reduced pressure to obtain **3** (12.8 g, 95%) as a yellowish oil, which was used for the following reaction without further purification. ^1^H NMR (400 MHz, CD_3_OD): δ 4.73 (dd, *J* = 9.0, 4.5 Hz, 2H), 3.29 (dd, *J* = 14.0, 4.5 Hz, 2H), 3.00 (dd, *J* = 14.0, 9.0 Hz, 2H), 2.02 (s, 6H). ^13^C{H} NMR (100 MHz, CD_3_OD): 172.2, 172.0, 51.6, 39.5, 21.0. [*α*]_D_^20^ = −99.5 (*c* = 1.01, D_2_O); lit.^30^ [*α*]_D_^20^ = −102.95 (*c* = 1.06, D_2_O). The chemical–physical data are in accordance with the compound reported in the literature.^30^

#### *S*,*S*′-((((2*R*,2′*R*)-3,3′-Disulfanediylbis(2-acetamidopropanoyl))bis(azanediyl))bis(ethane-2,1-diyl)) Diethanethioate (**5**)

To a solution of *N*,*N*′-diacetyl-l-cystine (**3**) (6 g, 18.5 mmol), *S*-acetyl cysteamine trifluoroacetate salt (**4**) (8.6 g, 55.6 mmol), and HATU (21.1 g, 55.6 mmol) in DMF (93 mL) was added DIPEA (22.4 mL, 129.6 mmol). After stirring at room temperature for 16 h, the mixture was diluted with CHCl_3_ (500 mL) and washed with 5% aqueous LiCl (2 × 250 mL). The aqueous phase was extracted with CHCl_3_ (1 × 150 mL). The combined organic phases were washed with 1 N HCl (2 × 200 mL), H_2_O (1 × 200 mL), 5% aqueous NaHCO_3_ (2 × 200 mL), and brine (1 × 200 mL). The organic layer was dried over anhydrous Na_2_SO_4_, and the solvent was evaporated under reduced pressure to obtain **5** (6.13 g, 63%) as a white solid, which was used for the following reaction without further purification. A portion was purified by flash chromatography (CHCl_3_/MeOH, 98:2). ^1^H NMR (400 MHz, CD_3_OD): δ 4.66 (dd, *J* = 8.5, 5.5 Hz, 2H), 3.38 (m, 4H), 3.17 (dd, *J* = 14.0, 5.5 Hz, 2H), 3.03 (t, *J* = 6.5 Hz, 4H), 2.92 (dd, *J* = 14.0, 8.5 Hz, 2H), 2.33 (s, 6H), 2.01 (s, 6H). ^13^C{H} NMR (100 MHz, CD_3_OD): δ 194.4, 170.6, 169.8, 81.3, 38.7, 37.5, 27.7, 26.4, 19.8; mp = (decomp.) >250 °C; [*α*]_D_^20^ = −53.1 (*c* = 0.16, MeOH); HRMS (ESI-TOF) *m*/*z*: [M + H]^+^ calcd for C_18_H_31_N_4_O_6_S_4_, 527.1121; found, 527.1126.

#### (*R*)-*S*-(2-(2-Acetamido-3-mercaptopropanamido)ethyl) Ethanethioate (**6**)

To a suspension of **5** (5.88 g, 11.2 mmol) and TCEP-HCl (3.36 g, 11.7 mmol) in MeOH (75 mL) was added buffer acetate at pH 4 (20.2 mL). After stirring at room temperature for 16 h, the mixture was diluted with H_2_O (30 mL) and extracted with CHCl_3_ (3 × 150 mL). The combined organic phases were washed with brine and dried over anhydrous Na_2_SO_4_, and the solvent was evaporated under reduced pressure. The residue obtained was crystallized with ethyl acetate/petroleum ether to give **6** (3.6 g, 61%) as a white solid, which was used for the following reaction without further purification. A portion was purified by flash chromatography (gradient from DCM/MeOH (98:2) to DCM/MeOH (96:4)). mp = 126–128 °C; ^1^H NMR (400 MHz, CDCl_3_) δ 7.17 (t, *J* = 5.5 Hz, 1H), 6.80 (d, *J* = 8.0 Hz, 1H), 4.65 (ddd, *J* = 8.0, 6.5, 4.5 Hz, 1H), 3.55–3.34 (m, 2H), 3.03 (t, *J* = 6.5 Hz, 2H), 2.99 (ddd, *J* = 14.0, 8.0, 4.5 Hz, 1H), 2.76 (ddd, *J* = 14.0, 10.0, 6.5 Hz, 1H), 2.35 (s, 3H), 2.06 (s, 3H), 1.60 (dd, *J* = 10.0, 8.0 Hz, 1H). ^13^C{H} NMR (100 MHz, CDCl_3_): δ 196.3, 170.4, 170.0, 54.2, 39.6, 30.7, 28.5, 26.8, 23.2; [*α*]_D_^20^ = −37 (*c* = 0.87, CHCl_3_); lit.^15^ [*α*]_D_^20^ = −40 (*c* = 0.87, CHCl_3_); HRMS (ESI-TOF) *m*/*z*: [M + H]^+^ calcd for C_9_H_17_N_2_O_3_S_2_, 265.0675; found, 265.0681. The chemical–physical data are in accordance with the literature.^15^

#### (*R*)-2-Acetamido-3-mercapto-*N*-(2-mercaptoethyl)propanamide (**1**)

To a solution of **6** (600 mg, 2.3 mmol) in H_2_O (11 mL) was added NH_4_OH (11 mL). After stirring at room temperature for 30 min, the mixture was acidified with 37% HCl (around 11 mL). The aqueous phase was extracted with CHCl_3_ (5 × 80 mL). The combined organic phases were washed with brine and dried over anhydrous Na_2_SO_4_, and the solvent was evaporated under reduced pressure. The solid was triturated with hexane to give **1** (393 mg, 77%) as a white solid. mp = 130–132 °C; ^1^H NMR (400 MHz, D_2_O): δ 4.58 (t, *J* = 6.0 Hz, 1H), 3.60–3.49 (m, 2H), 3.04 (d, *J* = 6.5 Hz, 2H), 2.80 (t, *J* = 6.5 Hz, 2H), 2.20 (s, 3H); ^1^H NMR (400 MHz, CDCl_3_): δ 6.71 (br s, 1H), 6.47 (br d, *J* = 7.5 Hz, 1H), 4.58 (ddd, *J* = 7.5, 7.5, 4.2 Hz, 1H), 3.56–3.41 (m, 2H), 3.09 (ddd, *J* = 14.0, 7.5, 4.0 Hz, 1H), 2.68 (ddd, *J* = 14.0, 10.2, 7.5 Hz, 1H), 2.73–2.67 (m, 1H), 2.09 (s, 3H), 1.73 (dd, *J* = 10.2, 7.5 Hz, 1H), 1.43 (t, *J* = 8.5 Hz, 1H). ^13^C{H} NMR (100 MHz, CDCl_3_): δ 170.6, 170.1, 54.5, 42.5, 26.7, 24.3, 23.2; [*α*]_D_^20^ = −55 (*c* = 0.24, CHCl_3_); lit.^15^ [*α*]_D_^20^ = −50 (*c* = 1.2, CHCl_3_); HRMS (ESI-TOF) *m*/*z*: [M + H]^+^ calcd for C_7_H_15_N_2_O_2_S_2_, 223.0569; found, 223.0559. The chemical–physical data are in accordance with the literature.^15^

#### (*R*)-*N*-(6-Oxo-1,2,5-dithiazocan-7-yl)acetamide (**1**^ox^)

NACMEAA (**1**) (75 mg, 0.33 mmol) was dissolved in EtOAc (330 mL), the mixture was cooled at 0 °C, and then a solution of KHCO_3_ (45 mL, 10% w/v in water) was added. A solution of I_2_ (154 mg, 0.61 mmol) in EtOAc (10.5 mL) was added dropwise (the solution turned brown), and the mixture was stirred at the same temperature for 1 h. The reaction was quenched by the dropwise addition of aqueous sodium thiosulfate until the solution became colorless. The organic layer was separated, dried with Na_2_SO_4_, filtered, and concentrated to afford a white powder that was purified by flash chromatography eluting from pure CH_2_Cl_2_ to 5% MeOH in CH_2_Cl_2_, affording **1**^ox^ (55 mg, 75%) in four indistinguishable conformations.^24e^ mp = (decomp.) >250 °C; ^1^H NMR (400 MHz, DMSO-*d*_6_): δ conformer mixture 8.25 (br s, 0.5H), 8.16 (br s, 1H), 8.05 (br s, 0.5H), 7.68 (br s, 1H), 4.6–4.48 (m, 0.5H), 3.95–3.81 (m, 1H), 3.54–3.45 (m, 1H), 3.15–2.92 (m, 1H), 2.77–2.69 (m, 1H), 2.51–2.50 (m, 0.5H); ^13^C NMR (100 MHz, DMSO-*d*_6_) δ conformer mixture 174.2, 173.3, 170.6, 169.8, 169.5, 169.3, 52.5, 52.3, 48.7, 48.5, 29.4, 29.1, 23.0, 23.0, 22.8, 22.8; HRMS (ESI-TOF) *m*/*z*: [M + H]^+^ calcd for C_7_H_13_N_2_O_2_S_2_, 221.0413; found, 221.0414.

## Supporting Information Available

^1^H and ^13^C NMR spectra for all compounds and 2D NMR spectra for compound **1**; optimization of the amide coupling conditions; determination of thiol p*K*_a_ values and reduction potential for compound **1** (PDF)

## References

[ref1] Cleland W. W. Dithiothreitol, a New Protective Reagent for SH Groups. Biochemistry 1964, 3, 480–482. 10.1021/bi00892a002. 14192894

[ref2] aWhitesides G. M.; Lilburn J. E.; Szajewski R. P. Rates of thiol-disulfide interchange reactions between mono- and dithiols and Ellman’s reagent. J. Org. Chem. 1977, 42, 332–338. 10.1021/jo00422a034.

[ref3] aWalsh S. J.; Bargh J. D.; Dannheim F. M.; Hanby A. R.; Seki H.; Counsell A. J.; Ou X.; Fowler E.; Ashman N.; Takada Y.; Isidro-Llobet A.; Parker J. S.; Carroll J. S.; Spring D. R. Site-selective modification strategies in antibody–drug conjugates. Chem. Soc. Rev. 2021, 50, 1305–1353. 10.1039/D0CS00310G. 33290462

[ref4] Bednar R. A. Reactivity and pH dependence of thiol conjugation to N-ethylmaleimide: detection of a conformational change in chalcone isomerase. Biochemistry 1990, 29, 3684–3690. 10.1021/bi00467a014. 2340265

[ref5] Stevens R.; Stevens L.; Price N. C. The stabilities of various thiol compounds used in protein purifications. Biochem. Educ. 1983, 11, 70–70. 10.1016/0307-4412(83)90048-1.

[ref6] aTrotta P. P.; Pinkus L. M.; Meister A. Inhibition by dithiothreitol of the utilization of glutamine by carbamyl phosphate synthetase: evidence for formation of hydrogen peroxide. J. Biol. Chem. 1974, 249, 1915–1921. 10.1016/S0021-9258(19)42872-X. 4361832

[ref7] aHeld K. D.; Biaglow J. E. Mechanisms for the oxygen radical-mediated toxicity of various thiol-containing compounds in cultured mammalian cells. Radiat. Res. 1994, 139, 15–23. 10.2307/3578727. 8016303

[ref8] aSingh R.; Whitesides G. M. A reagent for reduction of disulfide bonds in proteins that reduces disulfide bonds faster than does dithiothreitol. J. Org. Chem. 1991, 56, 2332–2337. 10.1021/jo00007a018.

[ref9] aLukesh J. C. III; Palte M. J.; Raines R. T. A Potent, Versatile Disulfide-Reducing Agent from Aspartic Acid. J. Am. Chem. Soc. 2012, 134, 4057–4059. 10.1021/ja211931f. 22353145 PMC3353773

[ref10] Mthembu S. N.; Sharma A.; Albericio F.; de la Torre B. G. 2-(Dibenzylamino)butane-1,4-dithiol (DABDT), a Friendly Disulfide-Reducing Reagent Compatible with a Broad Range of Solvents. Org. Lett. 2019, 21, 10111–10114. 10.1021/acs.orglett.9b04106. 31774687

[ref11] The Mitsunobu reaction requires two activating chemicals—one explosive—and generates two byproducts, one of which is toxic.

[ref12] aAwoonor-Williams E.; Rowley C. N. Evaluation of Methods for the Calculation of the pK_a_ of Cysteine Residues in Proteins. J. Chem. Theory Comput. 2016, 12, 4662–4673. 10.1021/acs.jctc.6b00631. 27541839

[ref13] BLDpharm-Germany 1000 g, EUR 73.

[ref14] aFraternale A.; Zara C.; Di Mambro T.; Manuali E.; Genovese D. A.; Galluzzi L.; Diotallevi A.; Pompa A.; De Marchis F.; Ambrogini P.; Cesarini E.; Luchetti F.; Smietana M.; Green K.; Bartoccini F.; Magnani M.; Crinelli R. I-152, a supplier of *N*-acetyl-cysteine and cysteamine, inhibits immunoglobulin secretion and plasma cell maturation in LP-BM5 murine leukemia retrovirus-infected mice by affecting the unfolded protein response. Biochim. Biophys. Acta – Mol. Basis Dis. 2020, 1866, 165922 10.1016/j.bbadis.2020.165922. 32800945

[ref15] Oiry J.; Mialocq P.; Puy J.-Y.; Fretier P.; Dereuddre-Bosquet N.; Dormont D.; Imbach J.-L.; Clayette P. Synthesis and Biological Evaluation in Human Monocyte-Derived Macrophages of *N*-(*N*-Acetyl-l-cysteinyl)-S-acetylcysteamine Analogues with Potent Antioxidant and Anti-HIV Activities. J. Med. Chem. 2004, 47, 1789–1795. 10.1021/jm030374d. 15027871

[ref16] Euerby M. R.; Partridge L. Z.; Gibbons W. A. Study of the chromatographic behavior and resolution of α-amino acid enantiomers by high-performance liquid chromatography utilizing pre-column derivatization with o-phthaldialdehyde and new chiral thiols. J. Chromatogr. A 1989, 239–252. 10.1016/S0021-9673(01)93125-5. 2745619

[ref17] Dombrowski A. W.; Aguirre A. L.; Shrestha A.; Sarris K. A.; Wang Y. The Chosen Few: Parallel Library Reaction Methodologies for Drug Discovery. J. Org. Chem. 2022, 87, 1880–1897. 10.1021/acs.joc.1c01427. 34780177

[ref18] Spears R. J.; McMahon C.; Chudasama V. Cysteine protecting groups: applications in peptide and protein science. Chem. Soc. Rev. 2021, 50, 11098–11155. 10.1039/D1CS00271F. 34605832

[ref19] Burns J. A.; Butler J. C.; Moran J.; Whitesides G. M. Selective reduction of disulfides by tris(2-carboxyethyl)phosphine. J. Org. Chem. 1991, 56, 2648–2650. 10.1021/jo00008a014.

[ref20] BLDpharm-Germany 100 g, EUR 223.

[ref21] Benesch R. E.; Benesch R. The Acid Strength of the -SH Group in Cysteine and Related Compounds. J. Am. Chem. Soc. 1955, 77, 5877–5881. 10.1021/ja01627a030.

[ref22] Gambardella G.; Cattani G.; Bocedi A.; Ricci G. New Factors Enhancing the Reactivity of Cysteines in Molten Globule-Like Structures. Int. J. Mol. Sci. 2020, 21, 6949 10.3390/ijms21186949. PMC7555924 32971812

[ref23] *E*°′_βME_ = −0.260 V; see ref (2b). βME turned out to be the more appropriate reference compound, since in parallel redox equilibrium between equal amounts of NACMEAA and oxidized DTT, only trace amounts of NACMEAA were in the oxidized form.

[ref24] aCarugo O.; Cemazar M.; Zahariev S.; Hudaky I.; Gaspari Z.; Perczel A.; Pongor S. Vicinal disulfide turns. Protein Eng. 2003, 16, 637–639. 10.1093/protein/gzg088. 14560048

[ref25] aHawkins H. C.; Freedman R. B. The reactivities and ionization properties of the active-site dithiol groups of mammalian protein disulphide-isomerase. Biochem. J. 1991, 275, 335–339. 10.1042/bj2750335. 2025220 PMC1150057

[ref26] aDobson C.; Evans P.; Radford S. Understanding how proteins fold: the lysozyme story so far. Trends Biochem. Sci. 1994, 19, 31–37. 10.1016/0968-0004(94)90171-6. 8140619

[ref27] aArtymiuk P. J.; Blake C. F.; Rice D. W.; Wilson K. S. The structures of the monoclinic and orthorhombic forms of hen egg-white lysozyme at 6 Å resolution. Acta Crystallogr., Sect. B: Struct. Commun. 1982, 38, 778–783. 10.1107/S0567740882004075.

[ref28] Peters T. J. All about Albumin: Biochemistry, Genetics, and Medical Applications. San Diego: Academic Press; 1995.

[ref29] Kaichang L.; Yuan L. Modified protein adhesives and lignocellulosic composites made from the adhesives. Patent Appl. US 20,040,037,906 A1, 2004.

[ref30] Oba M.; Tanaka K.; Nishiyama K.; Ando W. Aerobic Oxidation of Thiols to Disulfides Catalyzed by Diaryl Tellurides under Photosensitized Conditions. J. Org. Chem. 2011, 76, 4173–4177. 10.1021/jo200496r. 21480642

