# Supplementary material for: Dithiol Based on l-Cysteine and Cysteamine as a Disulfide-Reducing Agent
Source: J Org Chem. 2022 Jul 21;87(15):10073–9. doi: 10.1021/acs.joc.2c01050 (PMC9361291; doi:10.1021/acs.joc.2c01050)

A Dithiol based on L-Cysteine and Cysteamine as a Disulfide-Reducing Agent

Francesca Bartoccini, Michele Retini, Rita Crinelli, Michele Menotta, Alessandra Fraternale, Giovanni Piersanti*

Department of Biomolecular Sciences, University of Urbino Carlo Bo
Piazza Rinascimento 6, 61029 Urbino, PU, Italy.
E-mail: giovanni.piersanti@uniurb.it
https://sites.google.com/uniurb.it/ giovannipiersanti

Supporting Information

Table of contents

1. Abbreviation S3
2. Table S1. Reaction Optimization for the amide couplings S4
3. Determination of thiol pK_a values of compound **1** S5
4. Reduction potential of compound **1** S6
5. Mapping redox-active disulfides in lysozyme and BSA S7
6. References S8
7. Copies of ^{1}H NMR and ^{13}C NMR spectra S9
8. COSY of compound **1** S15

Abbreviation

EDC, (1-Ethyl-3-(3-dimethylaminopropyl)carbodiimide hydrochloride); HOBt, 1-Hydroxybenzotriazole; HATU, O-(7-Azabenzotriazol-1-yl)-*N*,*N*,*N′*,*N′*-tetramethyluronium hexafluorphosphate; HBTU, *O*-(1*H*-Benzotriazol-1-yl)- *N,N,N′,N′*-tetramethyluronium hexafluorophosphate; HCTU, *O*-(6-Chlorobenzotriazol-1-yl)-*N*,*N*,*N′*,*N′*-tetramethyluronium hexafluorophosphate; PyBop, (Benzotriazol-1-yloxytripyrrolidinophosphonium hexafluorophosphate); COMU, (1-Cyano-2-ethoxy-2-oxoethylidenaminooxy)dimethylamino-morpholino-carbenium hexafluorophosphate; TCFH, Chloro-*N*,*N*,*N′*,*N′*-tetramethylformamidinium hexafluorophosphate; CDI, 1,1′-Carbonyldiimidazole; IBC, Isobutyl chloroformate; HOSu, *N*-Hydroxysuccinimide. TEA, Triethylamine; DIPEA, *N*,*N*-Diisopropylethylamine, NMI, *N*-Methylimidazole; NMM, *N*-Methylmorpholine; TCEP-HCl, (Tris(2-carboxyethyl)phosphine hydrochloride), NEM, *N*-ethylmaleimide, IAM iodoacetamide, ACN, acetonitrile and FA, formic acid.

Table S1. Reaction Optimization for the amide couplings

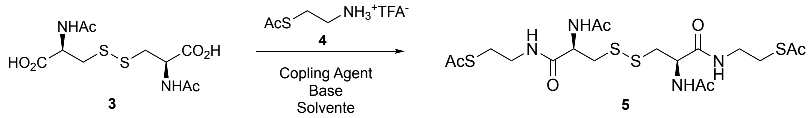

**Entry**	**Coupling Agent**	**Base**	**Solvent**	Yield of **5**
1	EDC/ HOBt	TEA	DMF	-
2	EDC/ HOBt	2,6-lutidina	5%TPGS-750M/H_2O	-
3	HATU	DIPEA	DMF	63%
4	HBTU	DIPEA	DMF	traces
5	HCTU	DIPEA	DMF	44%
6	PyBop	DIPEA	DMF	traces
7	COMU	DIPEA	DMF	traces
8	TCFH	NMI	ACN	-
9	TCFH	NMI	THF/ H_2O	-
10	CDI	-	THF	-
11	CDI	-	DMF	-
12	IBC, HOSu	NMM	EtOAc	-

Determination of thiol pKa values

The thiol pKa values of NACMEAA (**1**) were determined by measuring its absorbance at 238 nm at various pH. As reported by Benesch, the deprotonated thiolate absorbs much more strongly at 238 nm than does its protonated counterpart.[1] This attribute was exploited for determining thiol pKa values by Raines.[2] UV-Vis absorption spectra were recorded at 298.1 K on a Varian Cary-100 spectrophotometer equipped with a temperature control unit. The pH-metric UV-vis titration was carried out in water; the pH was adjusted by adding of NaOH. At least two sets of spectrophotometric titration curves were performed. pKa values were determined by fitting the data in **Figure S1** to eq 1,[8] which is derived from Beer's law and the definition of the acid dissociation constant.[2] In eq 1, C_T is total thiol concentration, ε_{SH}^{SH} is the extinction coefficient of the doubly protonated form, ε_{SH}^{S-} is the extinction coefficient of the singly protonated form, and ε_{S-}^{S-} is the extinction coefficient of the unprotonated form. The HypSpec computer program (HYPERQUAD package) was used to process the spectrophotometric data.[3] As a control, a pH titration of glutathione was also performed, giving a thiol pK_a of 9.0.

$$A_{238} = C_T \left(\frac{\varepsilon_{S-}^{S-}\, 10^{pH-pK_{a2}} + \varepsilon_{SH}^{S-} + \varepsilon_{SH}^{SH}\, 10^{pK_{a1}-pH}}{10^{pH-pK_{a2}} + 1 + 10^{pK_{a1}-pH}} \right) \qquad \text{(eq 1)}$$

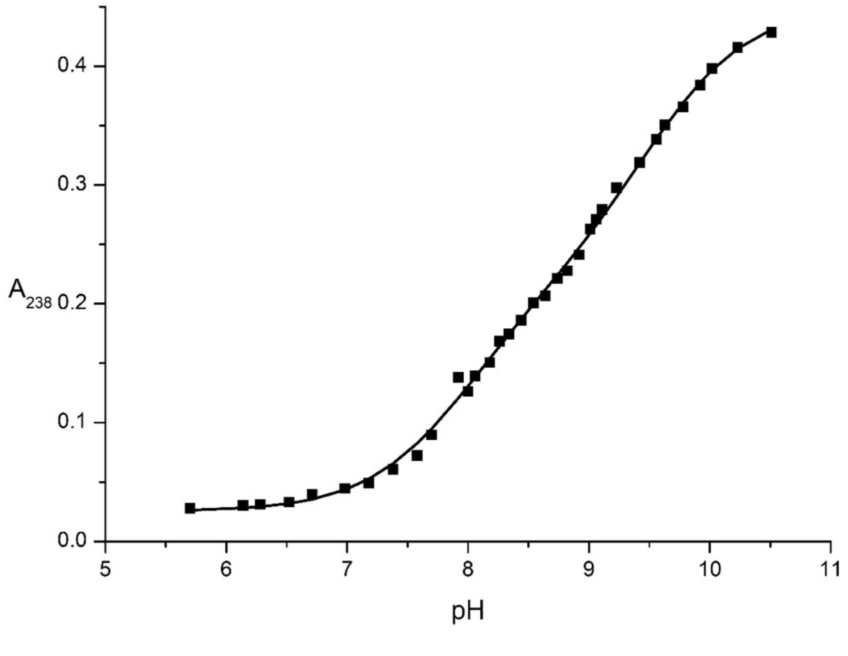

Figure S1. Plot of the Absorbances at 238 nm by varying the pH for the NACMEAA (**1**) ($7.0 \cdot 10^{-5}$ mol dm^{-3}). The fitting of the data yielded pK_a values of 8.0 (1) and 9.5 (1) with extinction coefficients of ε_{SH}^{SH} = 362 M^{-1} cm^{-1}, ε_{SH}^{S-} = 3133 M^{-1} cm^{-1}, ε_{S-}^{S-} = 6458 M^{-1} cm^{-1}. Values in parentheses are the standard deviations on the last significant figure.

Reduction potential of compound 1

The reduction potential ($E°'$) of **1** was obtained following the procedure described by Raines and coworkers.[4] The equilibrium constant of the reaction between **1** and oxidized BME (βME^{ox}) was determined by HPLC and used into a variation of the Nernst equation (eq 2).

$$E°'(\mathbf{1}) = E°'\ \beta\text{ME} - \frac{RT}{nF} ln \frac{[\mathbf{1}^{ox}][\beta\text{ME}]^2}{[\mathbf{1}][\beta\text{ME}^{ox}]} \qquad \text{(eq 2)}$$

Thiol-disulfide interchange equilibria were established between **1** and βME^{ox}. **1** (13.3 mg, 0.06 mmol) was added to a 25-mL round-bottom flask and flushed with N_2(g) for 30 min. A 50 mM stock solution of potassium phosphate buffer (pH 7) was degassed and purged with N_2(g) for 30 min immediately prior to use. Buffer (15 mL) was added followed by βME^{ox} (7.3 µl, 0.06 mmol) and the reaction mixture was stirred under N_2(g) for 24 h at room temperature.
The reaction mixture was then quenched by the addition of 3 N HCl (1:100 dilution) to prevent further reaction. An aliquot (0.1ml) of the quenched mixture was analyzed immediately by HPLC using column: Purospher® STAR RP-18 endcapped (5 µm) LiChroCART® 250-4 (25 cm x 4 mm); mobile phase, H_2O with 0.1% of FA and ACN: 0-2 min 2% ACN, 2-40 min 2-10% ACN; flow 0.8 ml/min. Compounds were detected by their absorption at 205 nm. Four peaks were evident in the chromatograms, and were anticipated to arise from **1**, **1^{ox}**, βME, and βME^{ox}. HPLC analysis of standard solutions revealed that the four peaks did indeed correspond to βME, (Rt = 5.98 min), βME^{ox} (Rt = 15.57 min), **1** (Rt = 16.58 min), and **1^{ox}** (Rt = 23.55. min). No evidence for mixed disulfides was apparent. To correlate peak area with concentration, calibration curves were developed for these compounds and were linear over the concentration range used (data not shown). From these calibration curves, the equilibrium concentration of each component was determined. Assuming that βME has $E°'$ = –0.26 V,[5] eq 2 (which is a variation of the Nernst equation) was used to calculate that **1** has $E°'$ = –(0.219 ± 0.004) V. This value is the mean ± SE from 10 experiments. The reverse reaction between **1^{ox}** and βME revealed that equilibrium had been established under the experimental conditions.

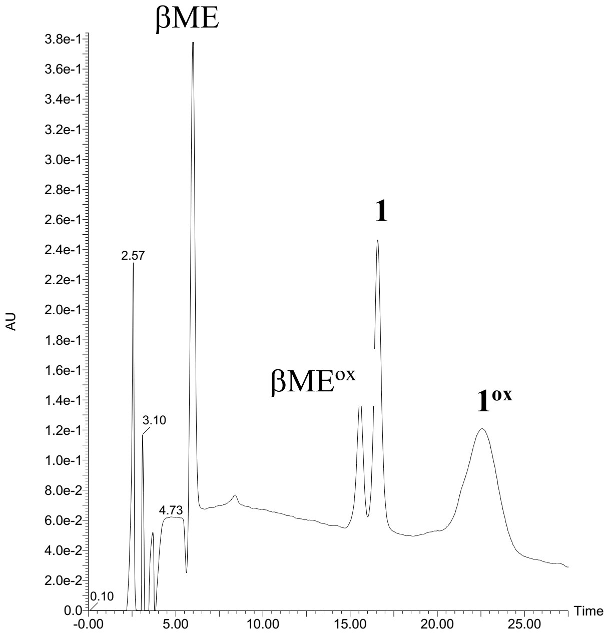

Figure S2.

Sample preparation for peptide mapping

Two micrograms of Lysozyme (Roche) or BSA (bovine serum albumin, Merck) in PBS were utilized for each experimental condition. Reduction was achieved by the addition of 0.5 mM or 1m M of **1**, expect for the Nontreated control sample (NR), and maintained at 37 °C for 30 min. Alkylation was performed by adding NEM or IAM to a final concentration of 4 mM in all samples at RT for 60 min in the dark. Peptides were then precipitated by acetone addition and overnight digested by trypsin/LysC mix in 25 mM ammonium bicarbonate at 37 °C. Peptides were then purified by C-18 Spin Colums (Pierce), dried and resuspended for MS analysis in 0.1% formic acid (FA) solution and quantified by BCA method (Pierce).

LC-MS/MS analysis of tryptic digests

Peptides were separated by an UltiMate 3000 nanoHPLC system (Thermo Scientific) coupled to an Orbitrap Exploris 240 Mass Spectrometer (Thermo Scientific). A total of 100 ng or 200 ng of peptides was separated by C-18 (Easy-Spray pepmap RSLC C18 2 µM, 15CMX50µM) reversed-phase chromatography using with a 60 minute gradient of 4–80% ACN containing 0.1% FA. MS/MS analysis was performed with a data-dependent Top20 method. For each cycle, a full MS scan (resolution: 120.000) in the Orbitrap with 3 million automatic gain control (AGC) target was followed by up to 20 MS/MS for the most intense ions. Maximum ion accumulation times were 30 ms for each full MS scan and 50 ms for MS/MS scans. Peptide sequences and modifications were extrapolated by SEQUEST algorithm considering, further the usual parameters, alkylation at cysteine by NEM (Δmass 125.0476Da) or by IAM (Δmass 57.021Da).

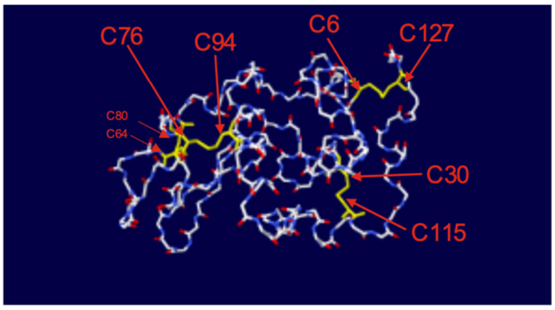

Figure S3. 3D structure of lysozyme

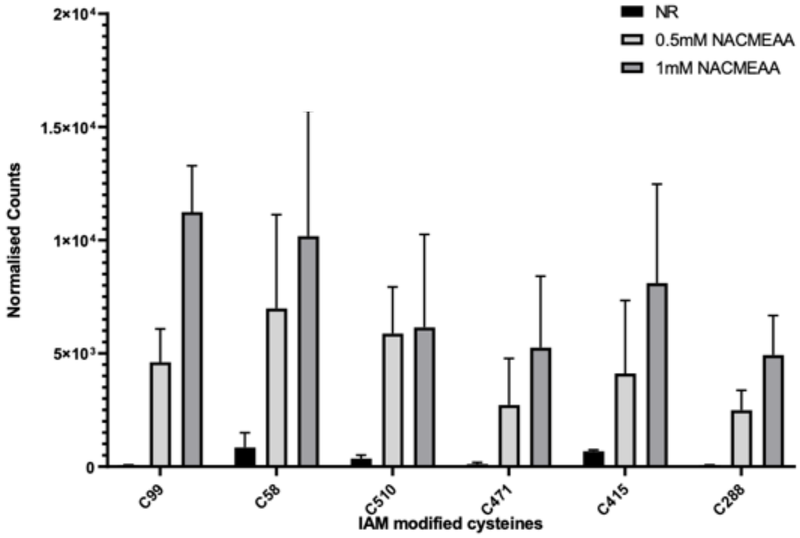

Figure S4. MS-identified peptides with cysteine modifications from BSA. Semi-quantitative analysis of cysteine-containing peptides obtained in nontreated control (NR) or treated with 0.5 mM or 1 mM NACMEAA and alkylated by IAM.

References

(1) Benesch, R. E.; Benesch, R. The Acid Strength of the -SH Group in Cysteine and Related Compounds. *J. Am. Chem. Soc.* **1955**, *77*, 5877–5881.
(2) Woycechowsky, K. J.; Wittrup, K. D.; Raines, R. T. A small-molecule catalyst of protein folding in vitro and in vivo. *Chem. Biol.* **1999**, *6*, 871–879.
(3) Gans, P.; Sabatini, A.; Vacca, A. Investigation of equilibria in solution. Determination of equilibrium constants with the HYPERQUAD suite of programs. *Talanta*, **1996**, *43*, 1739–1753.
(4) Woycechowsky, K. J.; Wittrup, K. D.; Raines, R. T. A small-molecule catalyst of protein folding in vitro and in vivo. *Chem. Biol.* **1999**, *6*, 871–879.
(5) Aitken, C. E.; Marshall, R. A.; Puglisi, J. D. An oxygen scavenging system for improvement of dye stability in single-molecule fluorescence experiments. *Biophys J.* **2008**, *94*, 1826–1835.

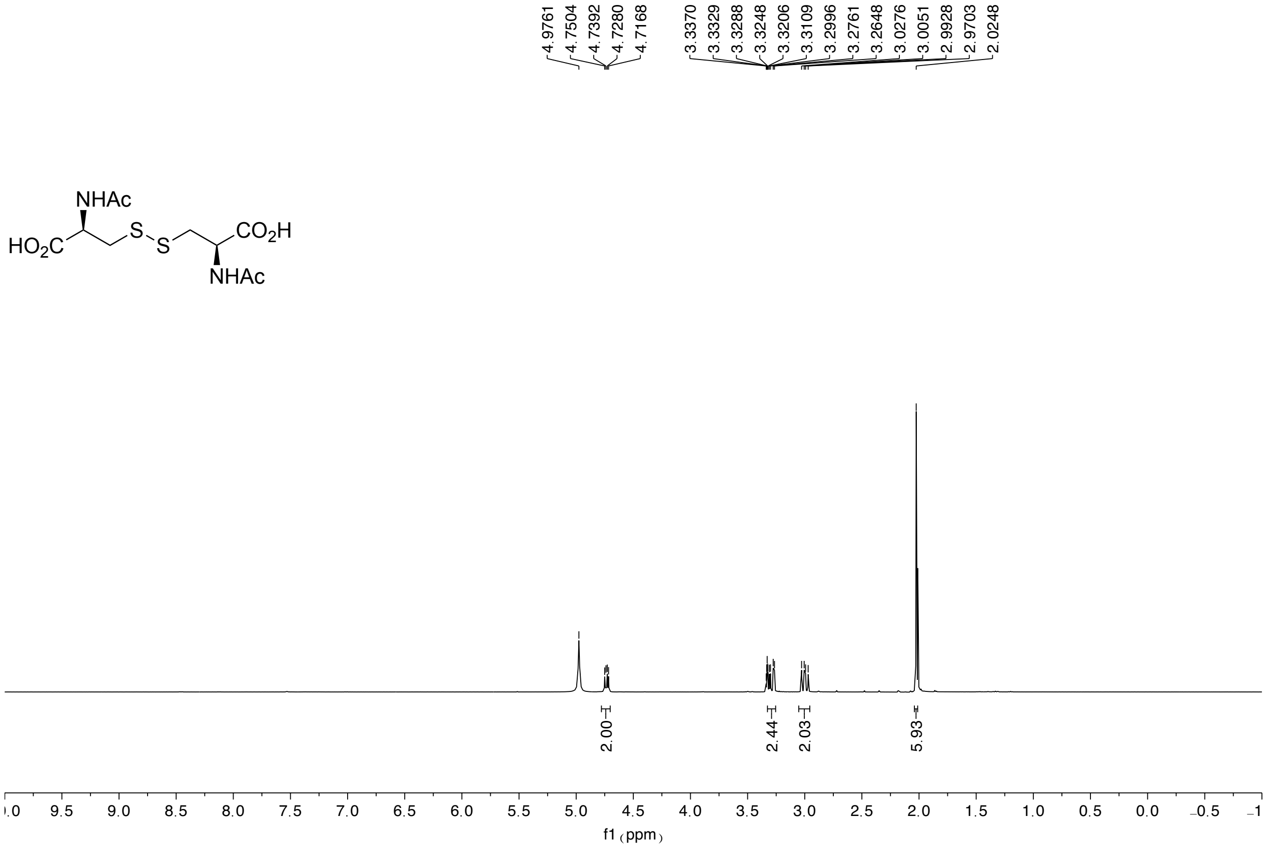

^{1}H NMR spectrum (400 MHz, CD_3OD) of compound 3

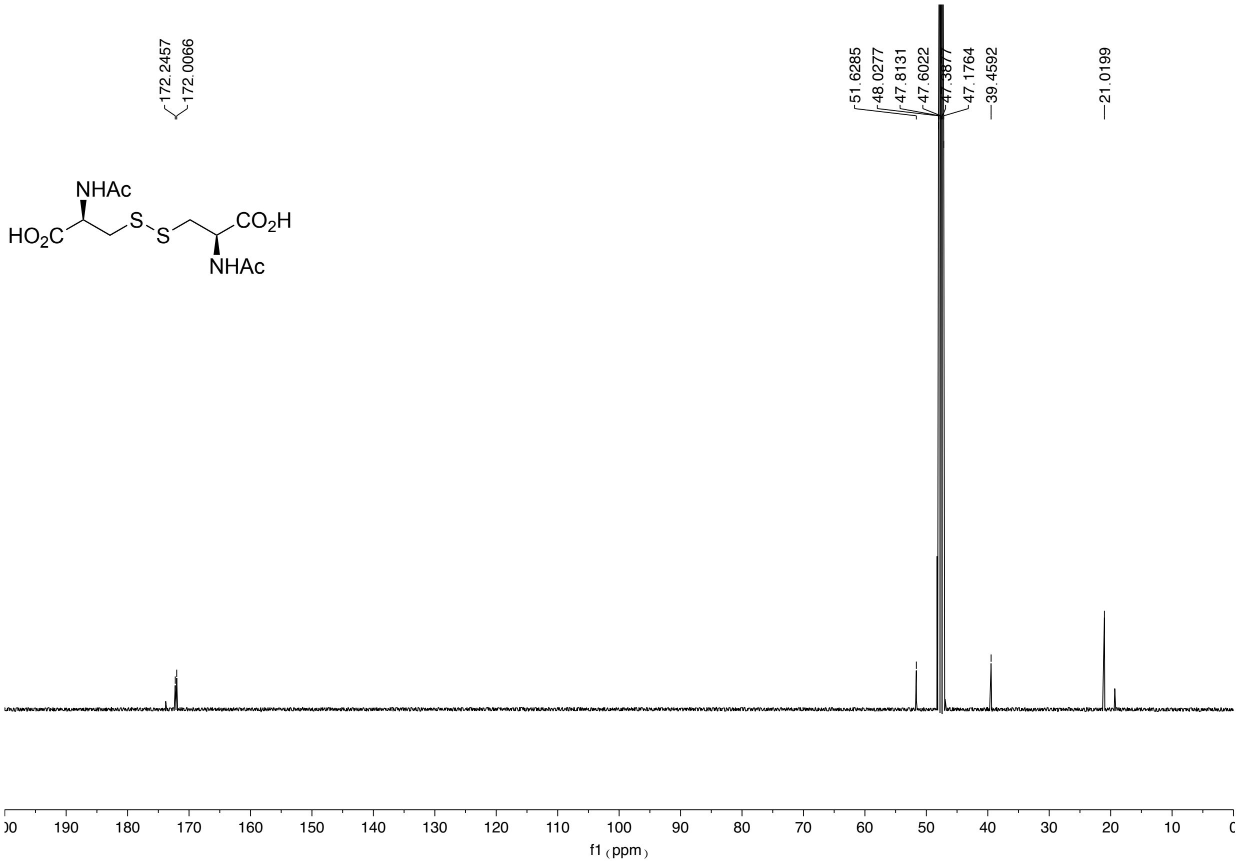

$^{13}C\{H\}$ NMR spectrum (100 MHz, CD_3OD) of compound 3

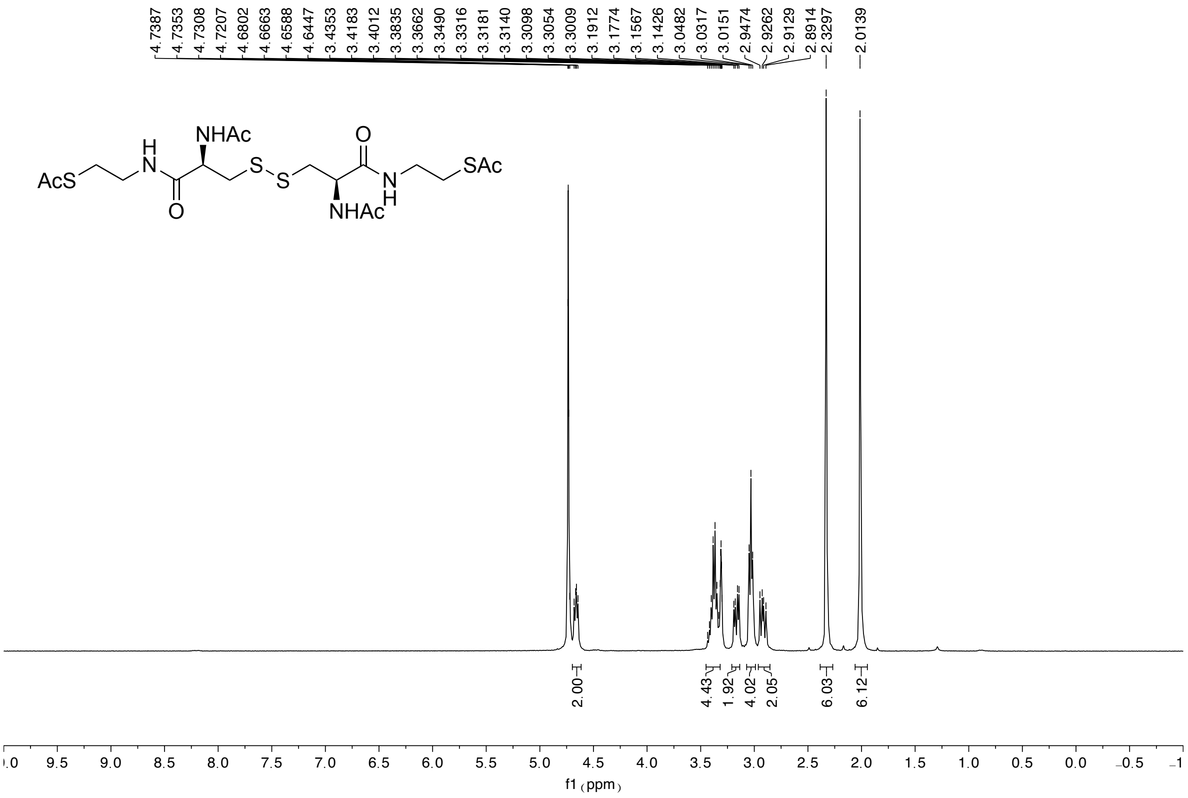

^{1}H NMR spectrum (400 MHz, CD_3OD) of compound 5

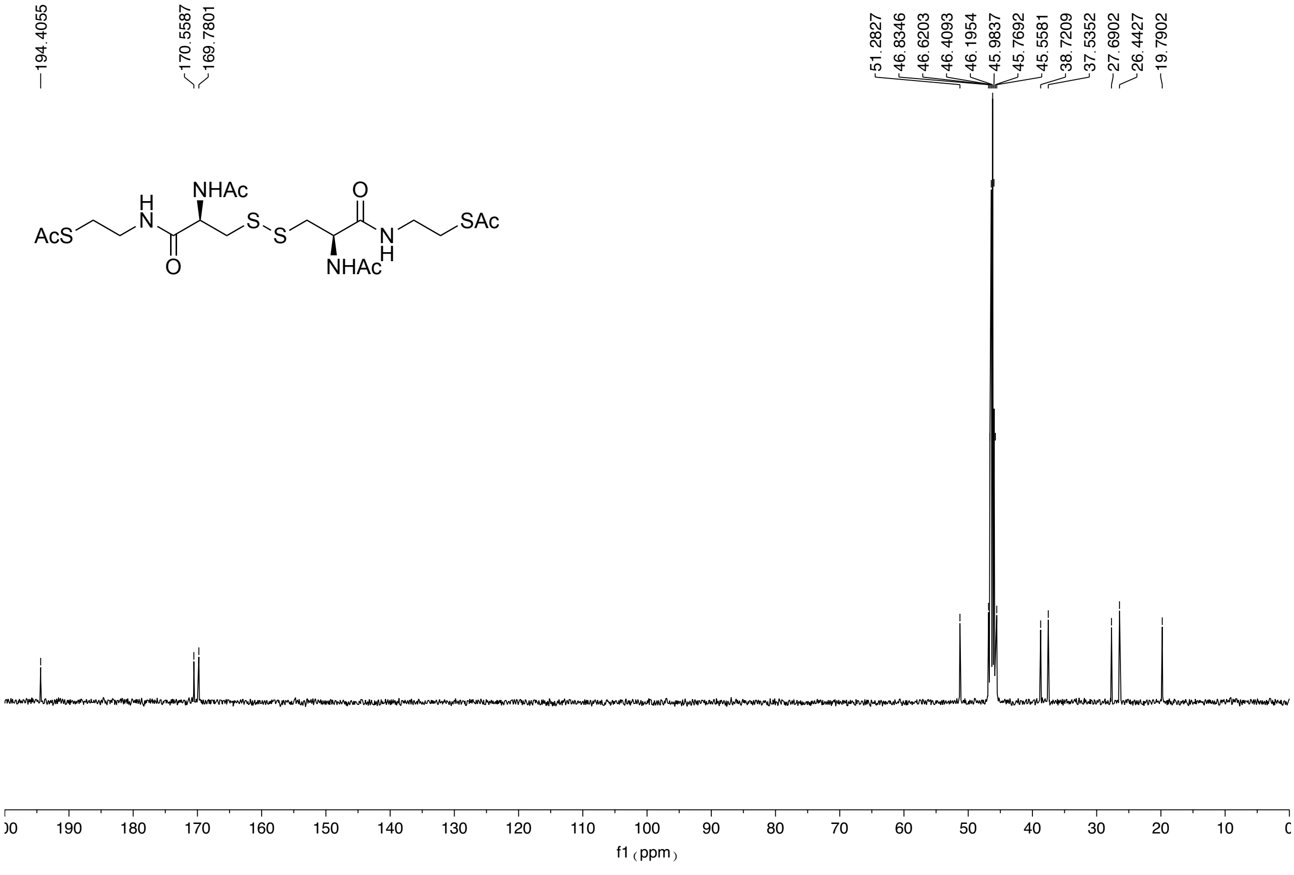

$^{13}C\{H\}$ NMR spectrum (100 MHz, CD_3OD) of compound 5

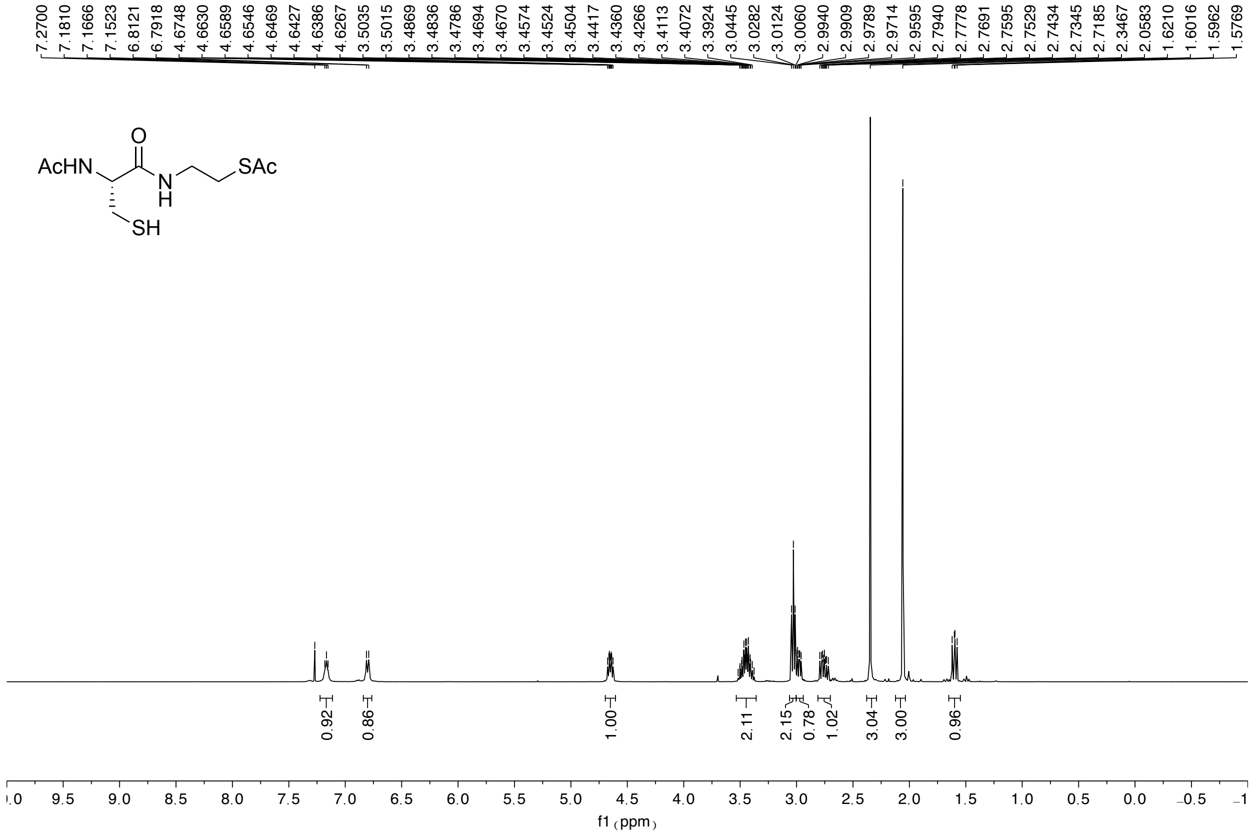

^{1}H NMR spectrum (400 MHz, $CDCl_3$) of compound 6

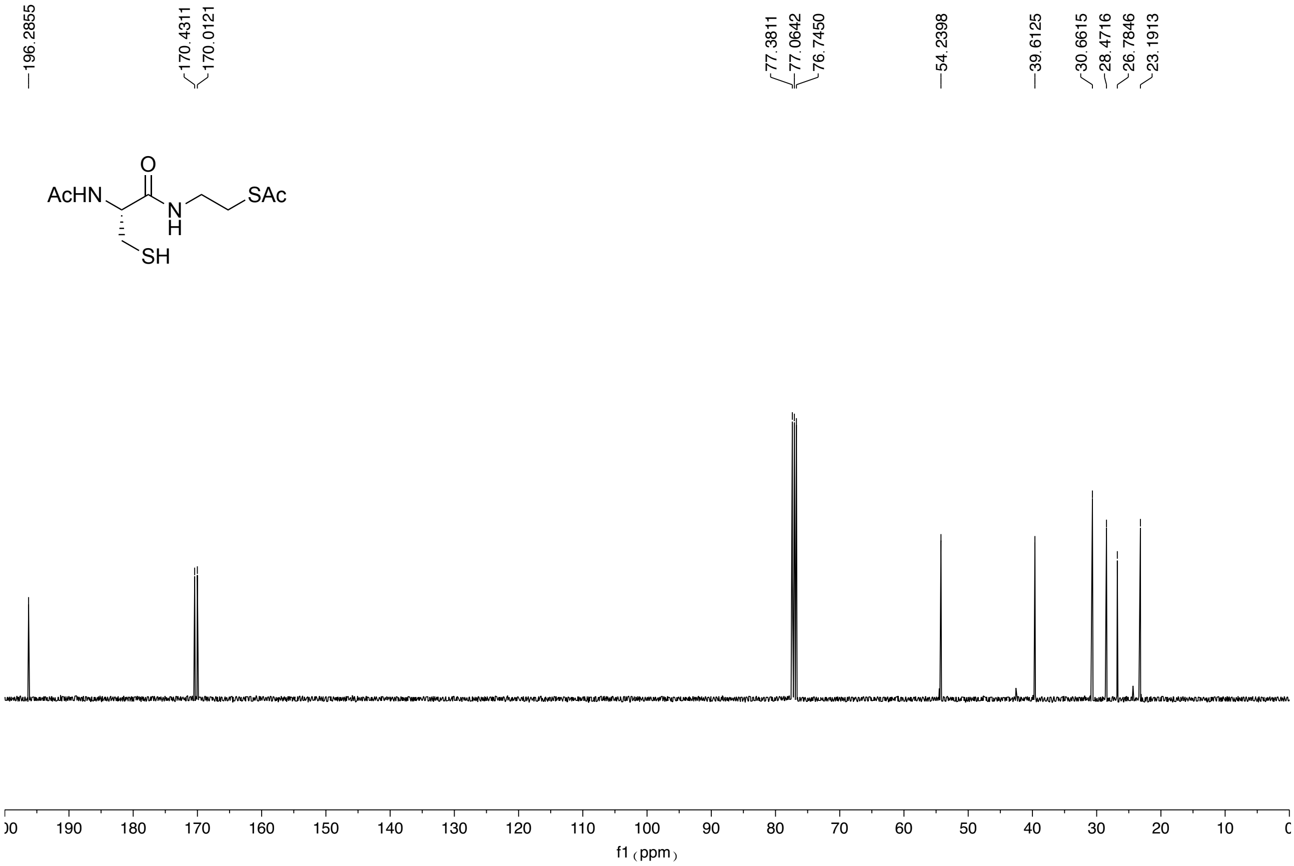

$^{13}C\{H\}$ NMR spectrum (100 MHz, $CDCl_3$) of compound 6

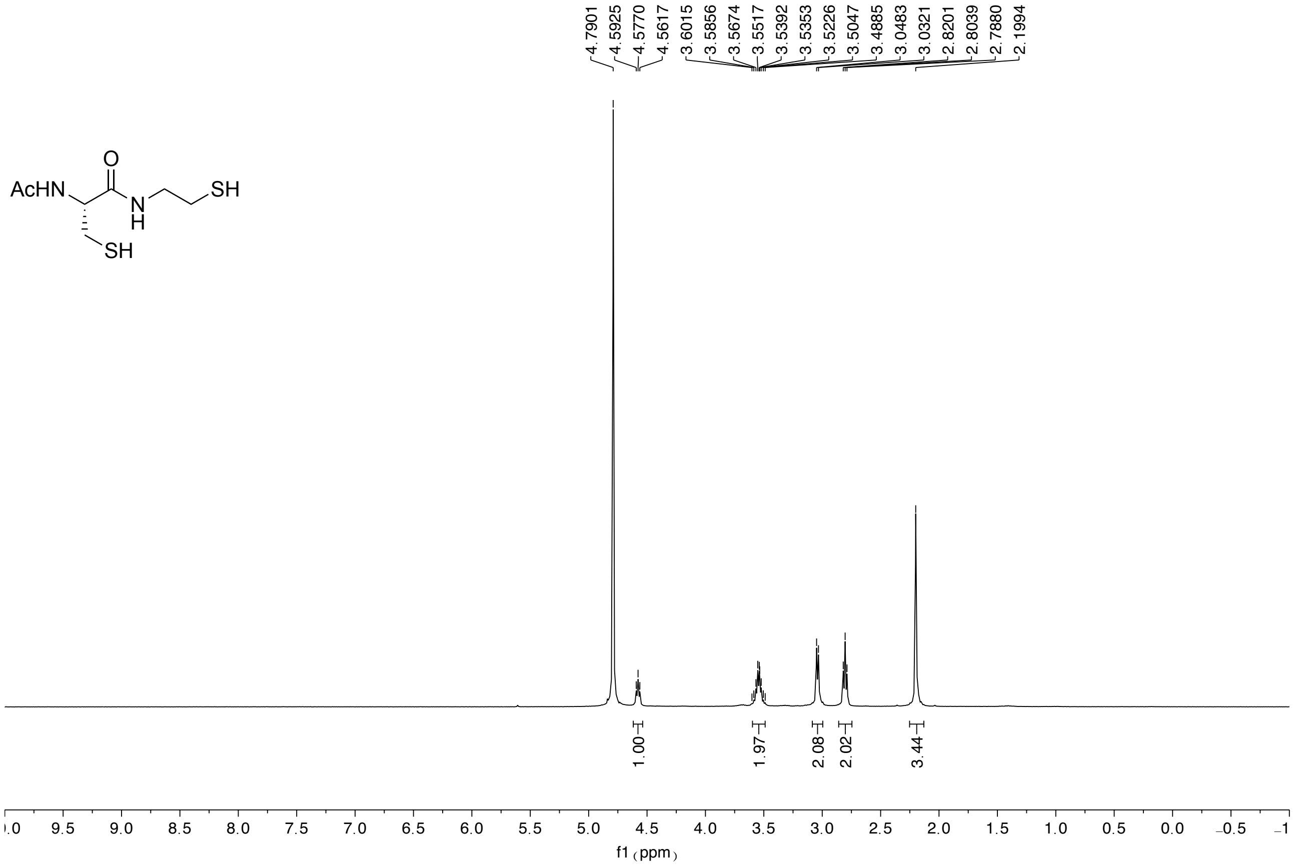

^{1}H NMR spectrum (400 MHz, D_2O) of compound 1

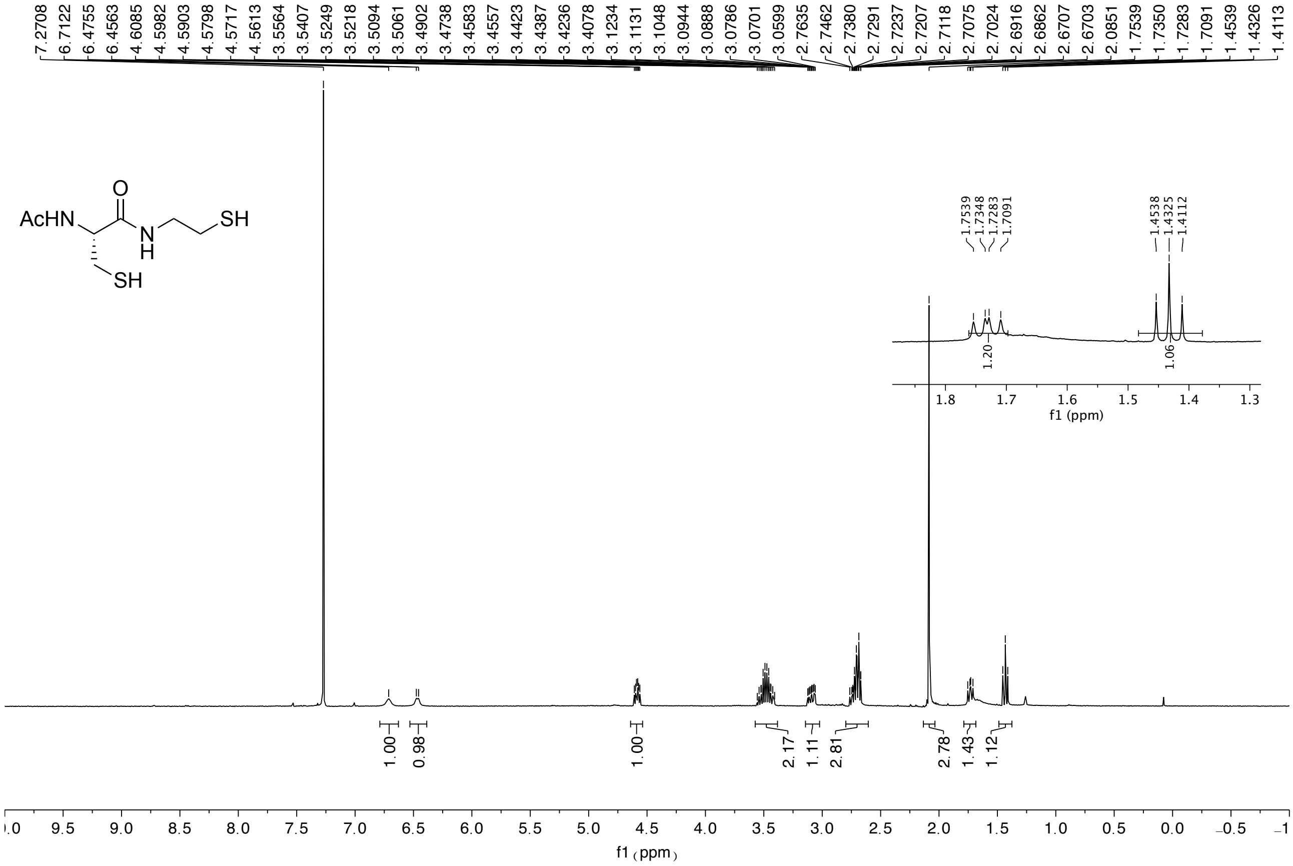

^{1}H NMR spectrum (400 MHz, $CDCl_3$) of compound 1

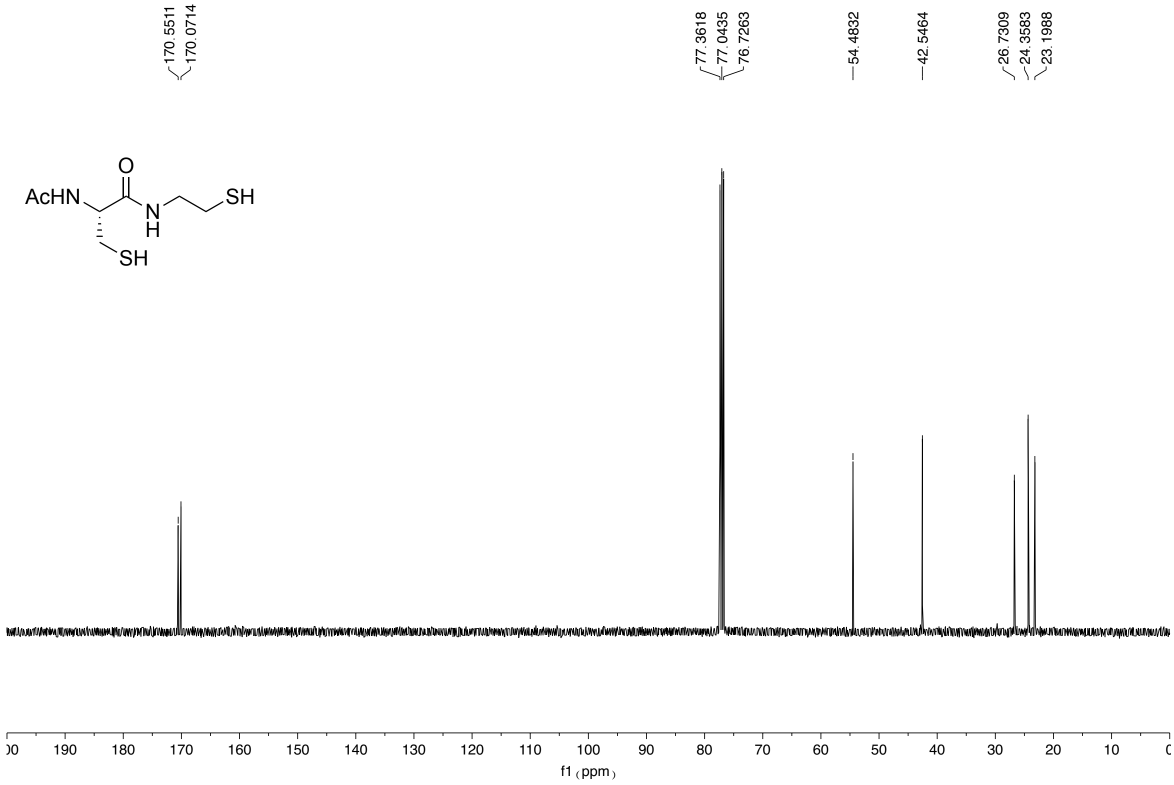

$^{13}C\{H\}$ NMR spectrum (100 MHz, $CDCl_3$) of compound 1

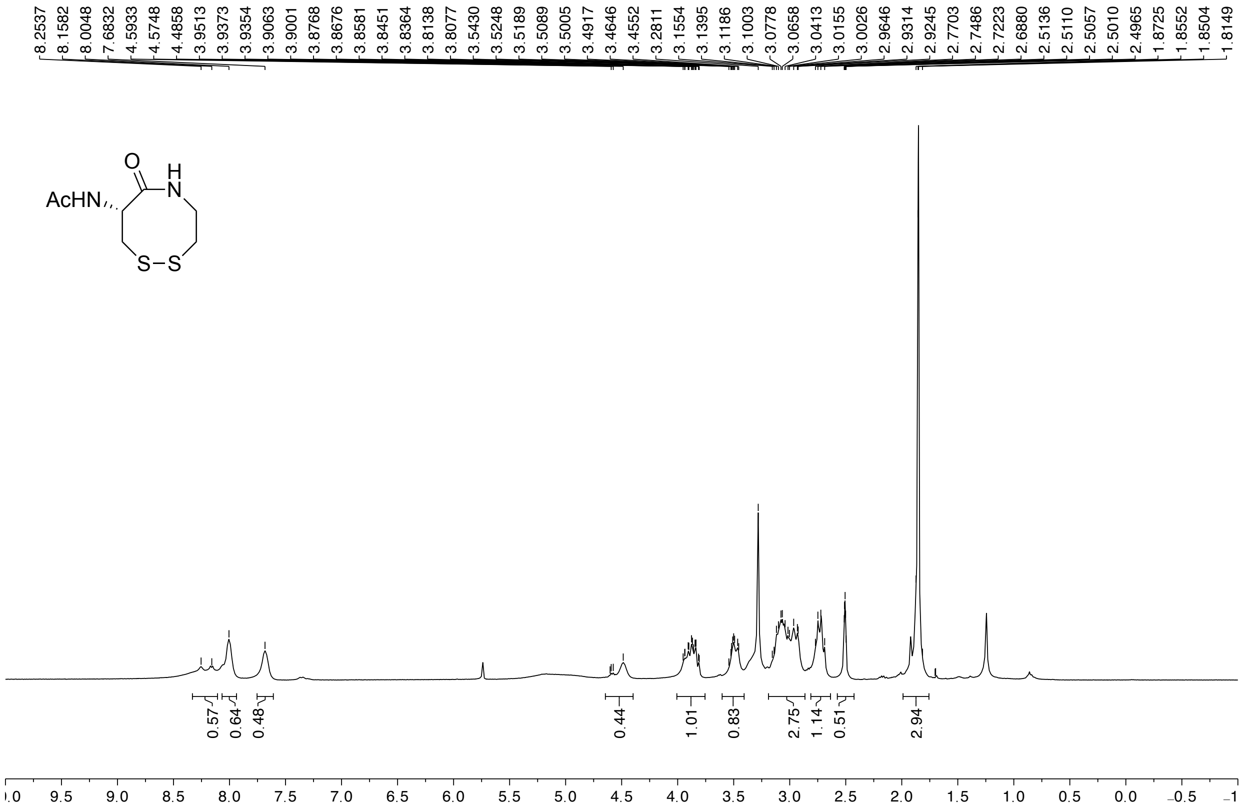

^{1}H NMR spectrum (400 MHz, DMSO-d_6) of compound 1^{ox}

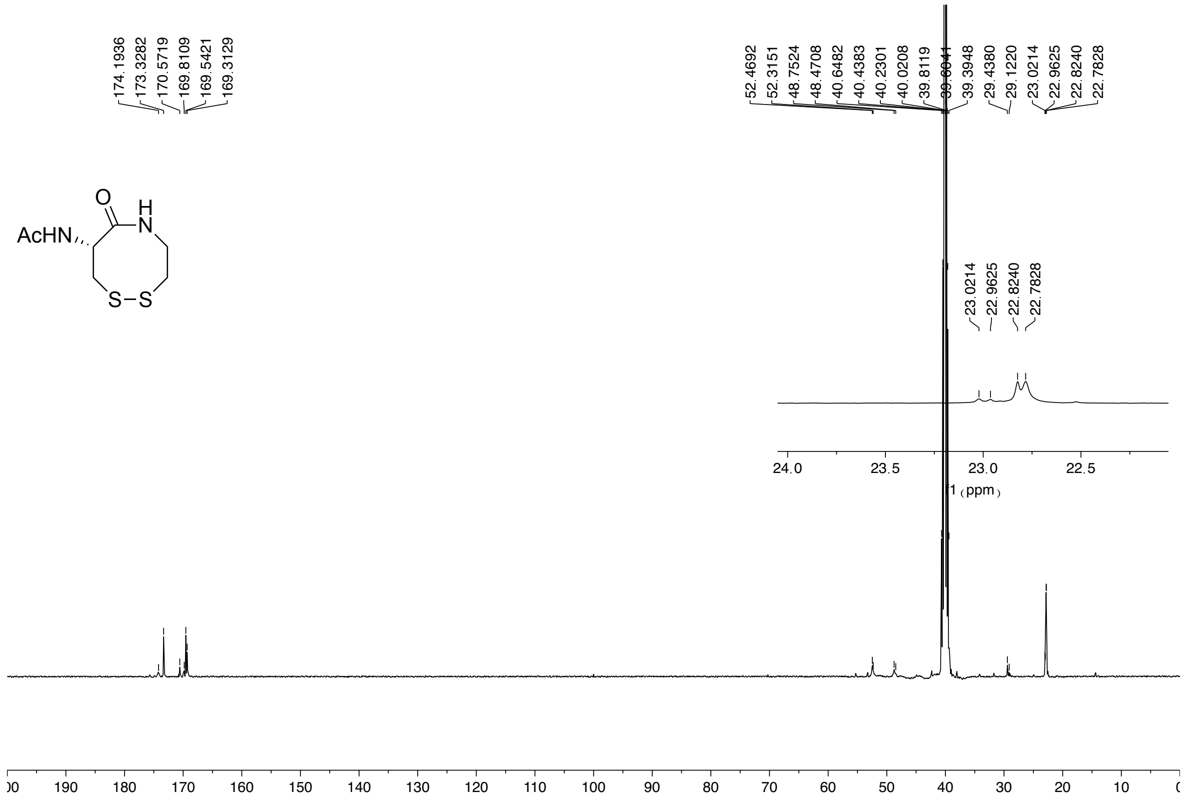

^{13}C{H} NMR spectrum (100 MHz, DMSO-d_6) of compound 1^{ox}

Figure S5. COSY of compound 1

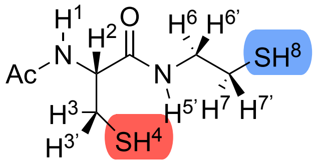

NACMEAA (**1**)

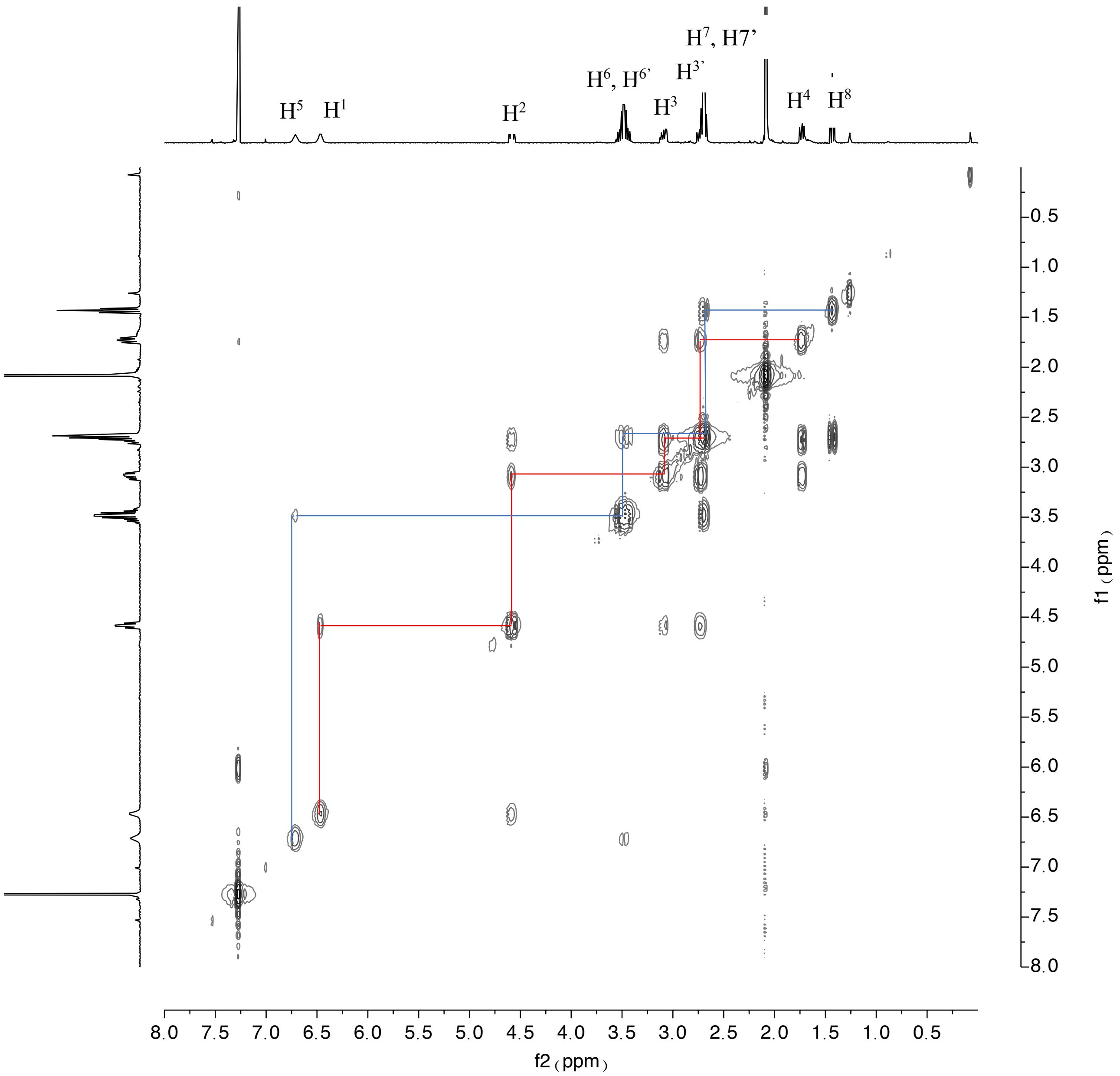

Supplement: Supplementary file 1 — jo2c01050_si_001.pdf [file jo2c01050_si_001.pdf]
